# Professor Marx's Tribute to Stieglitz

**Published:** 1847-04

**Authors:** 


### ART. XVIII.

*Zum Andenken an Dr. Johann Stieglitz, Königl. Hannoverschen Obermedicinalrath und Leibartz.* Von Dr. K. F. H. MARX, Hofrath und Professor in Göttingen.—*Göttingen,* 1846.

*A Tribute to the Memory of John Stieglitz, M.D., &c.* By Professor MARX, M.D., of Göttingen.—*Göttingen,* 1846. pp. 172.

THE Memoirs of Stieglitz by Professor Marx is a charming biographical *brochure,* and we are quite sure would have been freely used by Dr. Mackness in his work reviewed in another part of this Number, had he had the good fortune to see it entire before publishing his translation, for we believe the greater portion of its contents was unknown to him. We shall therefore notice it here, as a sort of supplement to our former article, and do not doubt that should Dr. Mackness's work attain to a second edition, he will find room for longer and more numerous extracts from it.

The Memoirs contain, first, the information supplied to Dr. Mackness; secondly, a series of extracts from the letters addressed by Stieglitz to his friend the Göttingen Professor; and then an estimate of his character. An Appendix contains some of his earlier reviews (referred to in the letters), two curious letters by Heim, and a fac-simile of Stieglitz's handwriting. Respecting the latter, we can truly say that it much more closely resembles the Arabic characters than those in use in Europe, and the Englishman must be well practised in German writing to decipher one-half of it.

Stieglitz was in extensive practice at Hanover. He was court-physician, chief of the medical police of the kingdom, obermedicinal-rath, &c. After Professor Marx had been three years at Göttingen, he paid a visit to Hanover, and called upon Stieglitz before eight o'clock in the morning: this first interview is thus described:—

"I found a man of an uncommon aspect; serious and friendly; his ample forehead showed the thinker. He sat enveloped in a cloud of tobacco-smoke, at a table covered with books and official documents, and drawing vigorously from time to time at his pipe. Our formal interview speedily changed into an animated conversation; but he was so discursive, and referred to such varied matters, that he touched upon men and things rather as if he were investigating than communicating. As we were interrupted, he pressed me cordially by the hand, and invited me to his table, that we might again resume the broken thread of our conversation. . . . . . . . The second visit, after the lapse of a few hours, was as if it were made to an old friend. Confidence was established, and an intellectual friendship cemented." (p. 17.)

From this time to his decease, a correspondence was kept up between the two friends. Extracts from the letters of Stieglitz are connected by a running commentary of the editor. We have marked a few passages as illustrating the everyday life and thoughts of the man. The following shows a crowded and defective state of the profession in Germany, or the writer is a *laudator temporis acti.*

"Towards the end of the last month I returned to Carlsbad; a journey I undertook for the sake of my wife, and not for my own pleasure or profit. . . . . . . . . I have become personally acquainted with from thirty to forty physicians, and have had consultation with many. The estimation of our calling, the predominant treatment of chronic diseases, and the power exercised over them by our art have excited in me many depressing thoughts. Everywhere I find physicians to be shrewd and adroit, well versed in the concerns of life and their modifying circumstances, but often filled with perverse and prejudiced ideas, taking no interest in science, unacquainted with the progress of medical science and practice, and not feeling the necessity of keeping up and renewing their earlier studies. Either old or new writers are read only by a very few. Very few have won my esteem." (p. 20.)

The medical journalism of Germany is also estimated lightly by Stieglitz. In one place he observes it is wonderful how rapidly the journals multiply and how wretched they are. In another he expresses a very indifferent opinion of the criticisms of the journalists. He was well acquainted with English writers, and was an extraordinarily rapid reader, and thought his time was as well spent on one of Sir Walter Scott's novels as on a book of mere facts.

Touches of worldly wisdom appear here and there; as the following on giving advice.

"Whoever has lived long in the world and been observant, has discovered that the giving of advice is dubious and insignificant. Every result is doubtful, and innumerable contingencies may happen. Who can know the external and internal condition of a person? In the long run every one takes that course to which his inclination leads him, or to which he is impelled by a good or evil genius." (p. 28.)

Or this, on faith in the future:

"Every one who has the power, and understands how to use and apply properly the present, can and must confide in the future. Sooner or later the fate of such a man takes a turn for the better, often in a quarter from which he least expects it, and important circumstances happen which elevate him." (p. 31.)

The treatment of incurable diseases:

"I often think some information is necessary as to the conduct of physicians with reference to incurable diseases—not as to what should be done, but what should be left undone.

"It often shocks me to witness extensive methods of cure propounded in steatoma, cancer, and innumerable other cases, in which it is well known no cure can be effected. A physician newly called in begins over again from the beginning what his predecessor has already practised. The constitution is shaken more and more, and the heroic remedies used can' have none other than evil results." (pp. 35-6.)

Danger of giving an unfavorable prognosis in incurable cases:

"The patient will often have a distinct answer from the physician, whether he has to apprehend death, and whether it be now unavoidable. Reasons are given to prove the importance of this information, and with what fortitude and resignation it will be received. All ground of hope must not be taken away. In Berlin an officer of artillery shot himself on the steps of Selle's house, after the latter, on being earnestly pressed, told the truth respecting his phthisis, and not waiting even long enough to get to his own residence. I witnessed a scene in the first year of the present century of quite another kind, which impressed upon me the propriety of never expressing the full truth in cases of this kind. A man will not suffer that to be plainly spoken by another, of which he is fully conscious himself, if it be evil and goes against himself. He will at times think of the possibility of the contrary." (p. 38.)

What is wisdom:

"It is wisdom not to intermingle with public business or intrude into it. I have extended this doctrine for a long time even to circumstances within my own province, and limit myself to giving an opinion when desired officially, and doing the work I am required, without taking further notice thereof, to see whether my advice has been followed or not, and bringing no personal influence to bear on the matter. When a man has become old and had much experience, he arrives at last at this passive behaviour and indifferentism, in contradiction to his character and early struggles. The mode in which public business is now conducted and the trade-like manner of most statesmen, must lead every one to this line of conduct who values his own peace or has it in view, or who is not prepared to sacrifice his self-respect." (pp. 43-4.)

We might go on multiplying quotations of this kind; we forbear, however, with a recommendation of this little work of Professor Marx's to all who take a pleasure in the perusal of German literature. The price is a trifle. We cannot, however, close this brief notice without the closing paragraph of the Memoirs. Writing of his conduct towards his patients and professional brethren, Marx observes:

"His name alone gave confidence to the suffering; his presence, comfort and help. Faith and trust preceded and followed him. Kind and serious, sympathizing and inquiring, he approached the patient; with comprehensive searching and kindly questionings he firmly assured him. That which he prescribed was the result of the most careful thought. Where it was possible he used the simpler and milder remedies; if not, those by which he could more decidedly attain the distinct and desired end.

"So he completed a busy and useful life; to the state a faithful servant, to society an ever ready helper; to his own people and his friends a sure rock of defence.

"His memory will never fade from their hearts. And in the spot where he so successfully laboured it will be a sacred duty to maintain it ever alive; for he was very much to many, although all did not know his full worth. But he who has once seen the glowing sunshine on the Alps,—the summits glittering—while on all around is the calm and gloom of night, will never have the same effaced from his memory." (p. 122.)

